# Genome-Wide Analysis and Identification of 1-Aminocyclopropane-1-Carboxylate Synthase (*ACS*) Gene Family in Wheat (*Triticum aestivum* L.)

**DOI:** 10.3390/ijms241311158

**Published:** 2023-07-06

**Authors:** Shuqing Liu, Chao Lei, Zhanhua Zhu, Mingzhen Li, Zhaopeng Chen, Wei He, Bin Liu, Liuping Chen, Xuejun Li, Yanzhou Xie

**Affiliations:** State Key Laboratory of Crop Stress Biology in Arid Areas and College of Agronomy, Northwest A&F University, Xianyang 712100, China; 18838054283@163.com (S.L.); lc18646032015@163.com (C.L.); 18864410385@163.com (Z.Z.); limingzhen995@163.com (M.L.); chenzp202202@163.com (Z.C.); hew0223@163.com (W.H.); binliu2022@163.com (B.L.); chenliupinghist@163.com (L.C.); xuejun@nwsuafedu.cn (X.L.)

**Keywords:** wheat, *ACS*, gene family, genome-wide, expression pattern

## Abstract

Ethylene has an important role in regulating plant growth and development as well as responding to adversity stresses. The 1-aminocyclopropane-1-carboxylate synthase (ACS) is the rate-limiting enzyme for ethylene biosynthesis. However, the role of the *ACS* gene family in wheat has not been examined. In this study, we identified 12 *ACS* members in wheat. According to their position on the chromosome, we named them *TaACS1*-*TaACS12*, which were divided into four subfamilies, and members of the same subfamilies had similar gene structures and protein-conserved motifs. Evolutionary analysis showed that fragment replication was the main reason for the expansion of the *TaACS* gene family. The spatiotemporal expression specificity showed that most of the members had the highest expression in roots, and all *ACS* genes contained W box elements that were related to root development, which suggested that the *ACS* gene family might play an important role in root development. The results of the gene expression profile analysis under stress showed that *ACS* members could respond to a variety of stresses. Protein interaction prediction showed that there were four types of proteins that could interact with TaACS. We also obtained the targeting relationship between *TaACS* family members and miRNA. These results provided valuable information for determining the function of the wheat *ACS* gene, especially under stress.

## 1. Introduction

Wheat is one of the most important crops globally, and the world’s wheat production in 2021 was about 770.88 million tons, as reported by the Food and Agriculture Organization of the United Nations (FAO) (https://www.fao.org/faostat/-en/#data/QCL/visualize accessed on 9 November 2022). The challenges of global climate change and population explosion are becoming increasingly serious. The stability of wheat yield is critical to ensure world food security and economic development [1]. Wheat yield and quality are seriously affected by a variety of biotic and abiotic stresses. The study of stress-related genes in wheat and their utilization in breeding can improve wheat stress resistance and reduce the effects of stress on wheat growth and development.

Ethylene is a major endogenous hormone in plants that play an important regulatory role in mechanical damage [2,3], biotic/abiotic stress [4,5], and growth and development, such as the process of flower development [6,7], fruit maturity [8,9], and root development [10,11,12]. There are two main steps for ethylene biosynthesis. The first step is that the substrate S-adenosyl methionine (SAM) generates ethylene precursor 1-aminocyclopropane-1-carboxylicacid (ACC) under the action of 1-aminocyclopropane-1-carboxylicacid synthase (ACS). The second step is that ACC is oxidized to ethylene, CO2, and cyanide under the action of the ACC oxygenate (ACO) [13]. Of these, the conversion process of SAM to ACC is the rate-limiting step in ethylene biosynthesis, which depends on ACS. Therefore, ACS is the key rate-limiting enzyme for ethylene synthesis in plants [14]. The current research has shown that the expression of *ACS* genes in plants can be induced by biotic or abiotic stress, which changes the ethylene content in plants, activates the ethylene-mediated signal pathway, and regulates the expression of downstream genes, thus improving the stress resistance of plants. Therefore, the *ACS* gene family has attracted the attention of increasing numbers of researchers. However, the functional studies of the *ACS* gene family in wheat have not been reported.

It has been shown that the ACS enzyme is a pyridoxal phosphate-dependent enzyme that belongs to the aminotransferase superfamily and has a multigene subfamily in higher plants [15]. Though ACS enzymes are a divergent multigene family, their primary structure has a similar molecular size (441 to 496 amino acids) and contains seven highly conversed regions [16,17]. Among them, region 5 is the PLP-binding site that is necessary for the *ACS* family. The N-terminal end of the ACS enzyme is a highly conserved region containing leucine and serine residues. The C-terminus is a hypervariable region that consists of 18 to 85 residues and is the core region when classifying subfamily members [18]. At present, with the release of genome data, members of the *ACS* gene family in many plants have been identified. For example, 12 *ACS* genes have been found in *Arabidopsis* [19], including at least 9 in tomato [20], 6 in rice [21], 13 in pumpkin [22], and 10 and 11 *ACS* genes in grapes and poplars, respectively [21]. To date, however, there have been no reports on the identification and analysis of *ACS* family members in wheat.

Previous studies have shown that *ACSs* play important roles in plant growth and development as well as in their response to biotic and abiotic stresses [23]. In *Arabidopsis*, *AtACS2* was related to lateral root development, and overexpressing *AtACS2* decreased the number of lateral roots significantly [24]. *AtACS2*, *AtACS6, AtACS7, AtACS8,* and *AtACS11* have been proven to be involved in pathogen invasion [25], and *AtACS6* has a high expression after injury [26]. In maize, the loss of *ZmACS6* expression has led to delayed leaf senescence under normal growth conditions, which enhances its drought resistance [27]. In addition, studies have shown that abiotic stresses, such as low temperature, drought, and hypoxia, could induce the expression of *ACS* in plants, thereby improving the stress resistance of plants. For example, after hypoxia stress treatment, the expression of *OsACS5* significantly increased in rice [28]. After the antisense, the *ACS* of carnation was introduced into tobacco through Agrobacterium tumefaciens, and the tolerance of transgenic tobacco to abiotic stress was significantly enhanced [29]. In cucumber, melon, watermelon, and pumpkin, the homologous genes *CmACS7*, *CsACS2*, *CitACS4,* and *CpACS27* affected sex differentiation by controlling flower development [30,31].

Therefore, in this study, we performed a genome-wide search and identification of the *TaACS* gene family. We systematically analyzed their chromosomal location, phylogenetic relationships, gene structure, collinearity among different species, promoter cis-elements, the protein interaction network, and miRNA target gene prediction. We also analyzed *TaACS* gene expression characteristics in different tissues and stresses. These results extended the study on the evolutionary history and biological functions of the *TaACS* gene family and provided an important reference for the subsequent screening of wheat resistance genes.

## 2. Results

### 2.1. Identification of the ACS Gene Family in Wheat

In order to identify the members of the *TaACS* gene family, we compared the amino acid sequences of *Arabidopsis* and rice *ACS* in the wheat protein database. The integrity of the *ACS* domain was confirmed using NCBI-CDD and SMART databases, which finally identified 12 *ACS* genes in wheat, which were named *TaACS1*-*TaACS12,* based on their positions in the chromosomes. The basic information of the *TaACS* gene family is listed in Table S1. The amino acid number of TaACSs was composed of at least 390 (*TaACS12*); at most 554 (*TaACS8*); the relative molecular weight (MW) was between 60.68 kDa (*TaACS12*) and 43.54 kDa (*TaACS11*); the isoelectric point (PI) ranged from 5.76 (*TaACS5*) to 9.11 (*TaACS12*); the distribution range of the Aliphatic index was between 77.59 and 88.13; and all the members of the *TaACS* gene family were exhibited as hydrophilic. Through the prediction of the subcellular location, it was found that most of the *TaACSs* were distributed in the nucleus, cytoplasm, and chloroplast.

### 2.2. Phylogenetic Analysis of the ACS Gene Family in Wheat

**Figure 1:**
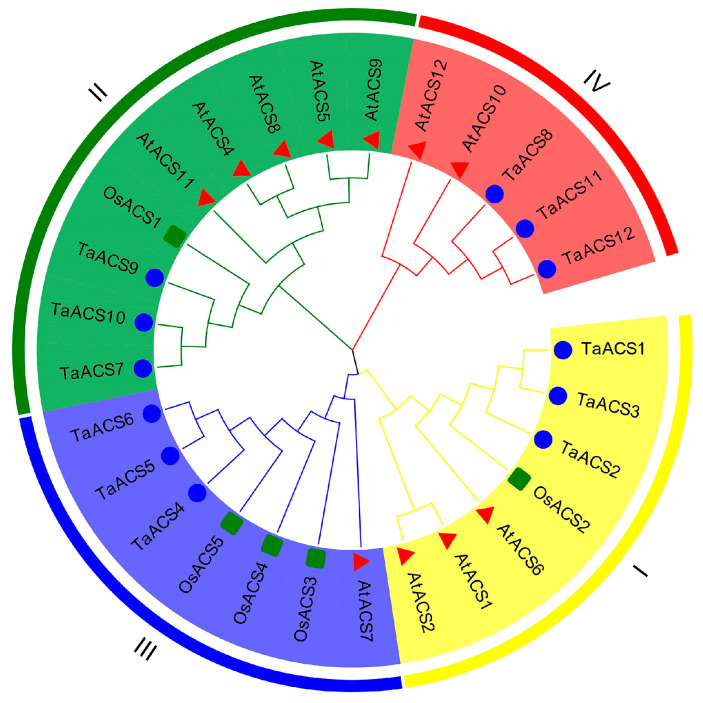
Phylogenetic tree of ACS proteins using the neighbor-joining method. The proteins of wheat (*Triticum aestivum* L.; Ta), rice (*Oryza sativa* L.; Os), and Arabidopsis (*Arabidopsis thaliana* L.; At) were indicated by different shapes. Different colors indicate different subfamilies (I–IV).

In order to understand the phylogenetic relationship, we constructed the phylogenetic tree using *Arabidopsis*, rice, and wheat proteins (Figure 1). The results showed that all 28 *ACS* genes were divided into four subfamilies according to the branches. Based on the classification of rice and *Arabidopsis*, we defined *TaACSs* to have the same branches as *Arabidopsis* and rice as subfamilies I-III. On the other hand, *TaACS8/11/12* and *AtACS10/12* without catalytic activity were clustered onto separate branches, which were called subfamily IV. We also compared and analyzed the protein sequences of 12 TaACS members, which showed that all members contained the typical seven conserved domains Box 1–7, which were conserved amino acid sequences at the N-terminal. By contrast, at the C-terminal, different subfamily members had significant differences in the sequences (Figure S1).

### 2.3. Analysis of Protein Conserved Motif and Gene Structure of ACS in Wheat

**Figure 2:**
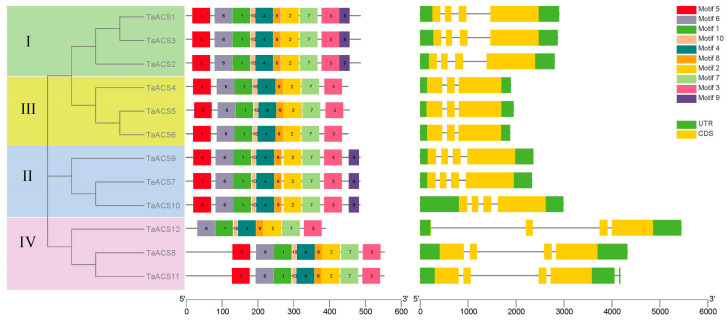
Phylogenetic relationships, conserved motifs, and gene structures of *ACS* genes in wheat. The different colors in conserved motifs represent the 10 identified motifs. The yellow and green rectangles in gene structures represent the coding sequences (CDSs) and untranslated regions (UTRs), respectively, and the black lines represent introns. The length of the CDS, UTR, and intron for each *ACS* gene is shown proportionally. I–IV is represented by green, yellow, blue, and red for different subfamilies.

The analysis of protein-conserved motifs and gene structures can provide important and valuable references when studying gene functions. There were 10 conserved motifs that were identified by the MEME website (Figure 2). Additionally, the members of the same subfamily contained similar conserved motifs. All TaACS proteins contained motifs 1, 2, 3, 4, 6, 7, 8, and 10. Compared to subfamilies I and II, all members of subfamily III lacked motif 9. In subfamily IV, TaACS8 and TaACS11 had one more motif 5 than TaACS12. An analysis of the gene structure showed that *TaACS12* and *TaACS6* had the longest and shortest gene lengths and intron lengths, respectively. The intron numbers of *TaACS* members were between one and four, and the exon numbers were between two and four. In the homologous gene, *TaACS7* and *TaACS9* had a longer 5’ UTR than *TaACS10*. In subfamily IV, compared to *TaACS8* and *TaACS11*, their homologous gene member *TaACS12* had a longer intron. In general, the more closely related members were, the more similar their gene structure was.

### 2.4. Chromosome Distribution and Collinearity Analysis of ACS Genes in Wheat

**Figure 3:**
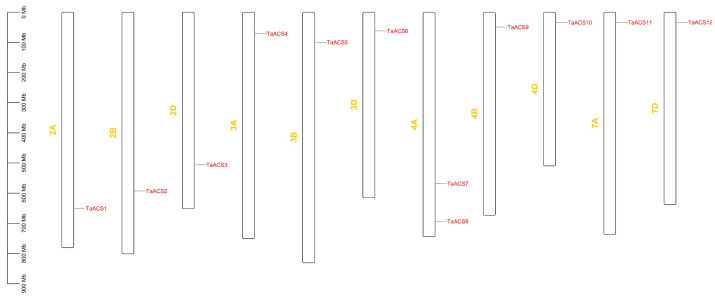
Relative positions of *TaACS* family members in wheat on chromosomes. All wheat chromosomes were drawn to scale based on their actual physical lengths.

**Figure 4:**
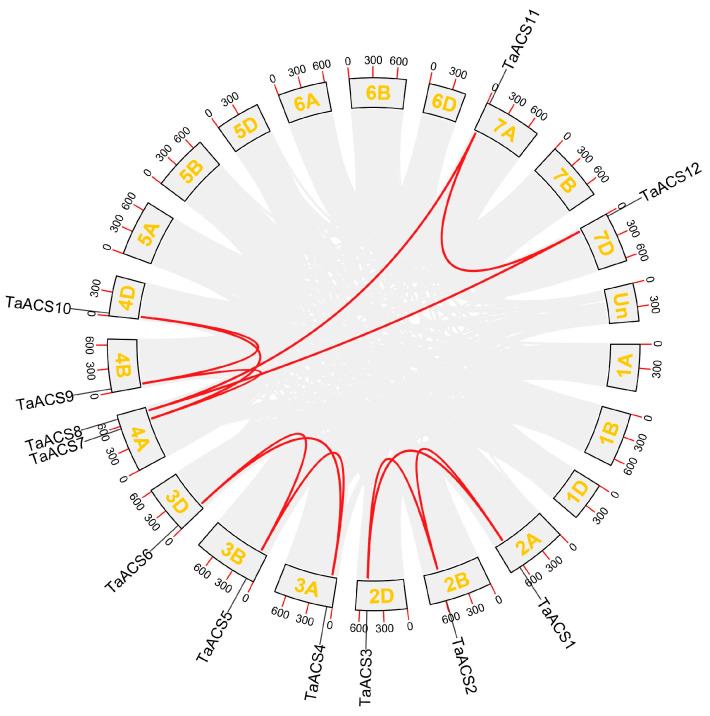
Distribution and duplication events of *TaACS* genes across the wheat genome. All *TaACS* genes were mapped to 21 wheat chromosomes in a circle using the Circos tool, and segmental duplications were mapped to their respective locations. Red lines represent segmental duplications. The chromosome numbers are marked outside of the circle.

The results of the chromosome localization visualization showed that *TaACS* genes were distributed on chromosomes 2A/2B/2D, 3A/3B/3D, 4A/4B/4D, and 7A/7D (Figure 3). There was only one member in each chromosome except for 4A Tandem duplication and segmental duplication, which are important modes for driving the evolution and amplification of plant genomes. In this study, we identified 12 segmental duplication genes, among which *TaACS8* and *TaACS11*(4A, 7A), *TaACS8* and *TaACS12*(4A, 7D) occurred between non-homologous chromosomes, and other segmental duplication gene pairs and occurred between homologous chromosomes (Figure 4). In order to study the evolutionary selection of *TaACS* genes, we calculated the Ka/Ks of segmental duplication and found that all Ka/Ks values were between 0.08 and 0.27, which was far less than one (Table 1), and indicated that the evolution of *TaACS* genes was mainly influenced by purifying the selection pressure to maintain their functional stability. In addition, we found that there were no tandem duplication events in this gene family. These results indicated that segmental duplication could play a major role in promoting the increase in the number of *ACS* genes in wheat.

**Figure 5:**
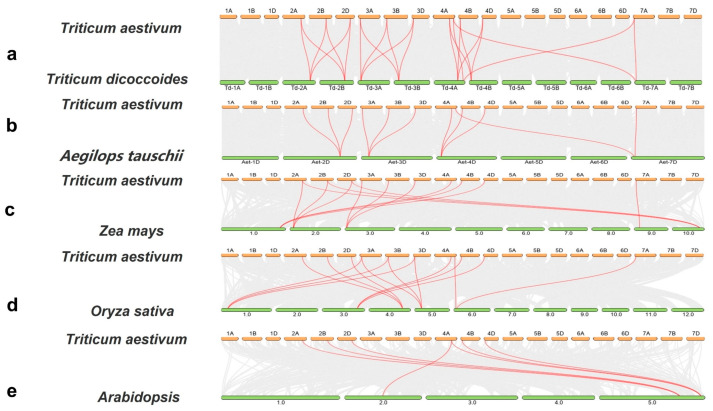
Synteny analysis of *ACS* genes between wheat and representative plant species (**a**–**e**). Gray lines in the background indicate the collinear blocks within wheat and other plant genomes, while the red lines highlight syntenic *ACS* gene pairs.

In order to further explore the evolution of the *TaACS* gene family, we analyzed the homologous relationships among *Triticum dicoccoides*, *Aegilops tauschii*, rice, maize, and *Arabidopsis*. The results showed that there were 22, 11, 14, 13, and 7 covariates between wheat and these five species, respectively (Figure 5). Among them, *TaACS1*-*TaACS11* was involved in the formation of homologous gene pairs in monocotyledons. Six members, *TaACS1/2/3* and *TaACS7/9/10* were involved in the formation of homologous gene pairs in dicotyledons. It was suggested that these genes might have a common genetic origin, and homologous pairs could exist before the differentiation of plant ancestors.

### 2.5. Analysis of Cis Elements in TaACS Gene Promoter

**Figure 6:**
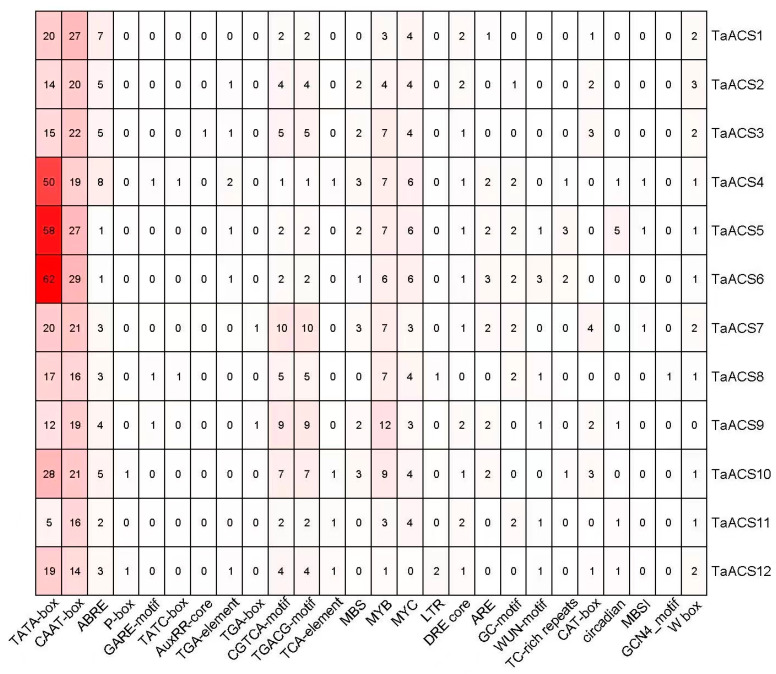
Cis-elements analysis of promoter sequences of *TaACSs*. The number in the cuboid indicates the number of these cis-acting elements, and the color shade can be positively related to the number contained.

In order to obtain a better understanding of the transcriptional regulation mechanism and potential function of these genes, we submitted a 2000 bp genomic sequence that was upstream of the transcription initiation site to the PlantCARE database to predict and analyze the cis-elements in the promoter. Subsequently, we collated cis-elements relating to hormones, stress, growth, and development except for the basic core elements of promoters, including TATA-box and CAAT-box (Figure 6). The results showed that there were 10 kinds of elements related to hormone response, such as the abscisic acid (ABA) response element ABRE, the gibberellin (GA) response elements P-box, the GARE-motif, and TATC-box, the auxin (IAA) response elements Auxin-core, TGA-element and TGA-box, the methyl jasmonate (MeJA) response elements CGTCA-motif and TGACG-motif, and salicylic acid (SA) response element TCA-element. There were nine kinds of elements related to stress, including MBS, MYB, and MYC (response to drought stress), LTR (response to low-temperature stress), DRE (response to low temperature, dehydration, and salt stress), ARE and GC motifs (response to hypoxia), WUN-motif (response to wound), and TC-rich repeats (response to defense and stress). The five types of elements related to growth and development, CAT box, MBSI, GCN4-motif, W box, and circadian (involved in meristem expression, flavonoid synthesis, endosperm expression, lateral root formation regulation, and circadian rhythm control), were also found in *TaACS* genes. All members, except for *TaACS12* and *TaACS9*, contained MYC and W box, suggesting that *TaACS* genes might play important regulatory roles in drought stress and lateral root formation. There were some members that had multiple elements, such as 10 CGTCA-motifs, 9 TGACG-motif elements in *TaACS9,* and 8 ABRE in *TaACS4*, respectively, which suggested that these genes could play important roles in ABA and MeJA signaling pathways. In general, the *TaACS* gene family may be widely involved in hormone metabolism and stress responses. 

### 2.6. Analysis of miRNA Targeting TaACS Gene

**Figure 7:**
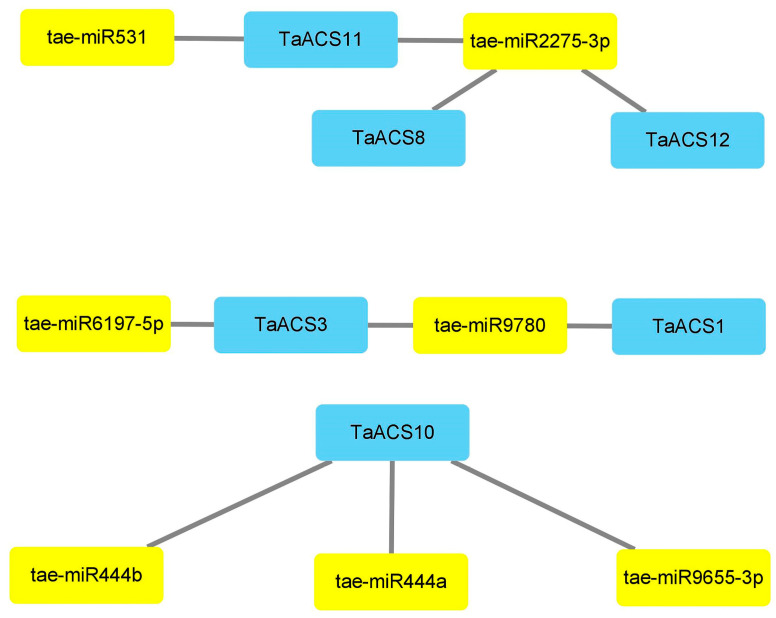
Analysis of miRNA targeting *TaACS* genes in wheat. Blue represents members of the *TaACS* family and yellow represents targeted miRNAs.

MicroRNAs (miRNAs) are small endogenous non-coding RNA molecules consisting of approximately 21-25 nucleotides. Predicting the targeting relationship between miRNA and *TaACS* is possible to further understand the potential function of *TaACS*. The results showed that there were 10 pairs of targeting relationships between 7 miRNAs and 6 TaACS members (Figure 7). Some members were regulated by multiple miRNAs, and some miRNAs were capable of regulating multiple members. Tae-miR2275-3p targeted three *TaACS* members (*TaACS8/11/12*). *TaACS10* was simultaneously targeted by tae-miR444b, tae-miR444a, and tae-miR9655-3p. In addition, there were relationships that were a one-to-one moderation. *TaACS11* was targeted by Tae-miR531. These results indicated that there was a close relationship between miRNAs and *TaACS*.

### 2.7. Expression Profile of ACS Gene in Wheat

**Figure 8:**
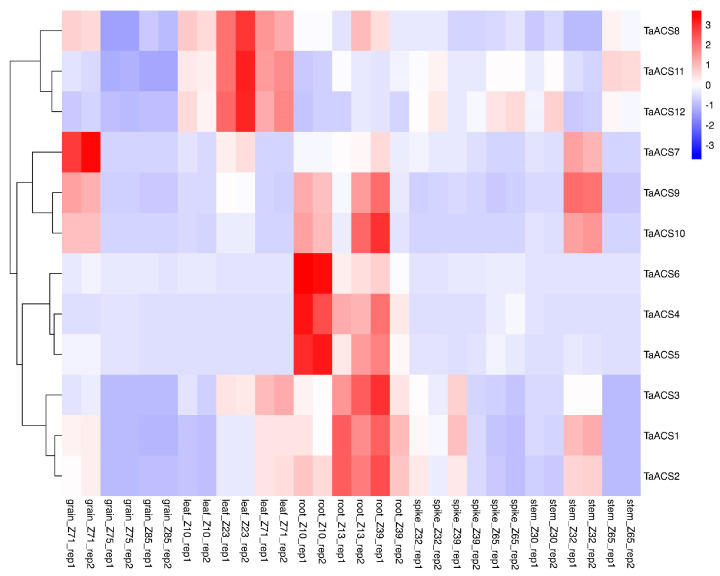
Expression profiles of *TaACS* genes in different tissues and organs. grain_Z71, grain_Z75, and grain_Z85 were 2d, 15d, and 30d grains after flowering, respectively. leaf_Z10, leaf_Z23, and leaf_Z71 represent the leaf at seedling stage, the flag leaf at the tillering stage, and leaf 2d after flowering, respectively. root_Z10, root_Z13, and root_Z39 represent roots at the seedling stage, three-leaf stage, and flag leaf stage, respectively. Spike_Z32, spike_Z39, and spike_Z65 represent the spikes at the two-edged stage, flag leaf stage, and flowering stage, respectively. stem_Z30, stem_Z32, and stem_Z65 were the ear at 1 cm length, the ear at two-edged stage, and the ear at flowering stage, respectively. The red, white, and blue cells represent the highest, medium, and lowest gene expression levels, respectively.

In order to understand the expression of the *TaACS* gene family in different periods and tissues, we obtained their expression profiles by RNA-Seq data (expVIP website). The original data were processed with log2 (tpm + 1) to obtain the expression heat map (Figure 8 and Table S3). The results showed that *TaACS1/2/3*, *TaACS4/5/6*, and *TaACS9/10* were highly expressed in roots at different stages; *TaACS8/11/12* were highly expressed in leaves at the tillering stage and second after flowering. *TaACS7* was highly expressed at the stem at the 2d grains after flowering and the ear at the two-edged stage. All *TaACS* had a low expression in the spike. Overall, most homologous genes had similar spatiotemporal expression patterns.

**Figure 9:**
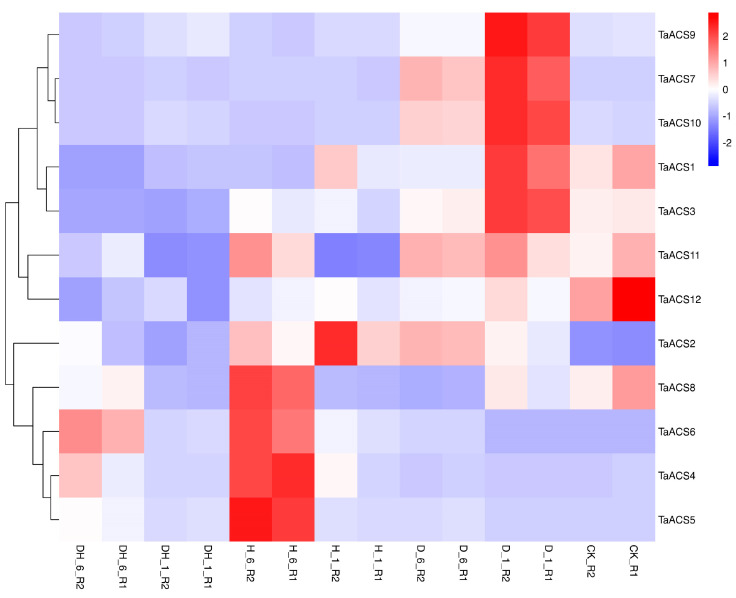
Expression profiles of typical *TaACS* genes in wheat before and after drought stress, heat stress, and co-drought and heat stress. D_1 and D_6 represent 1 h and 6 h after drought stress treatment of wheat, respectively; H_1 and H_6 represent 1 h and 6 h after hot stress treatment of wheat, respectively; DH_1 and DH_6 represent 1 h and 6 h after co-drought and heat stress treatment of wheat, respectively; CK represents no stress treatment of wheat. The red, white, and blue cells represent the highest, medium, and lowest gene expression levels, respectively.

**Figure 10:**
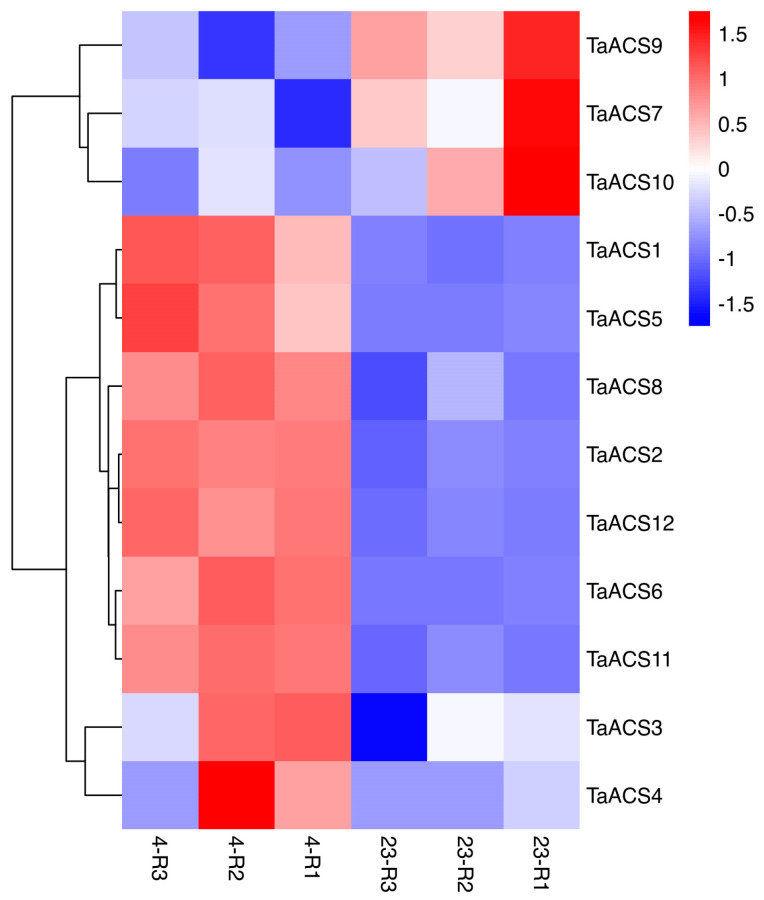
Expression profiles of typical *TaACS* genes in wheat before and after cold stress. 23 represents wheat without cold stress treatment; 4 represents wheat treated by 4 °C cold stress. The red, white, and blue cells represent the highest, medium, and lowest gene expression levels, respectively.

**Figure 11:**
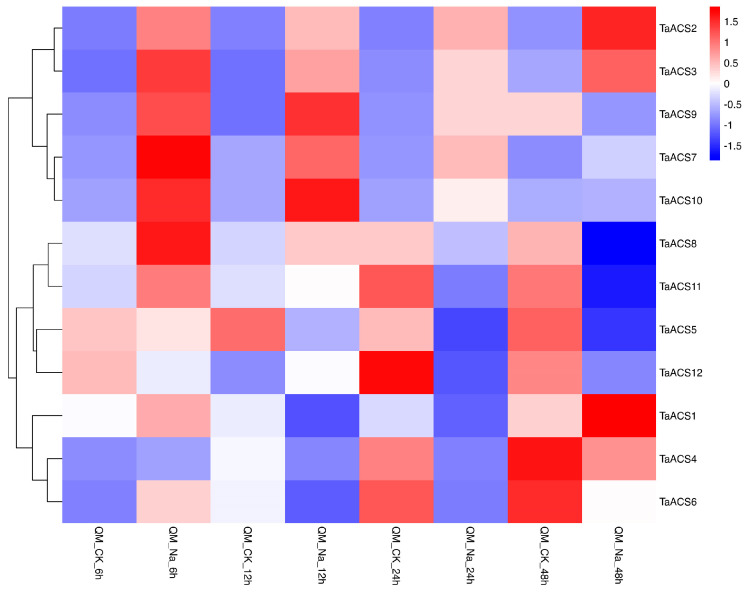
Expression profiles of typical *TaACS* genes in wheat treated with NaCl for 6 h, 12 h, 24 h, and 48 h. QM represents wheat “Qing Mai 6”; CK represents no stress treatment of wheat; Na represents NaCl treatment of wheat. The red, white, and blue cells represent the highest, medium, and lowest gene expression levels, respectively.

**Figure 12:**
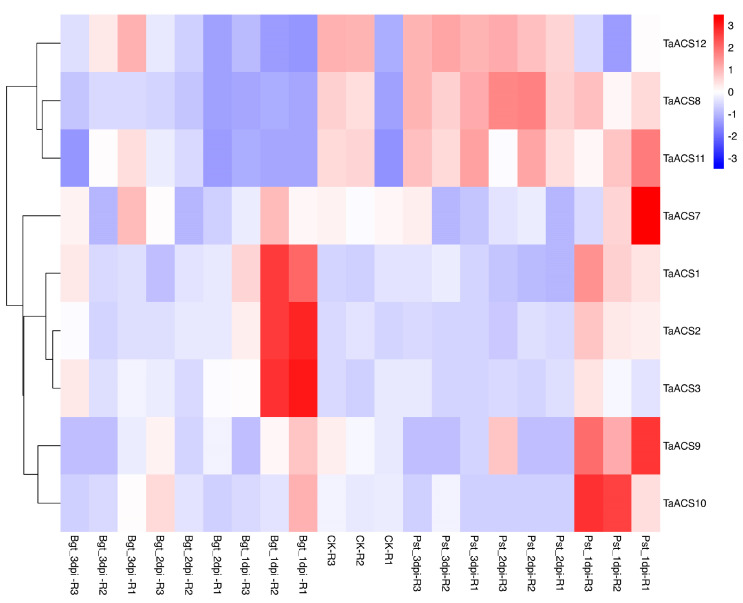
Expression profiles of typical *TaACS* genes in wheat before and after the injection of powdery mildew and stripe rust. Bgt_1dpi, Bgt_2dpi, and Bgt_3dpi represent 24 h, 48 h, and 72 h after powdery mildew pathogen was injected into wheat leaves; Pst_1dpi, Pst_2dpi, and Pst_3dpi represent 24 h, 48 h, and 72 h after stripe rust pathogen was injected into wheat leaves; CK represents leaves not injected with powdery mildew and stripe rust. The red, white, and blue cells represent the highest, medium, and lowest gene expression levels, respectively.

ACS is a key enzyme in the biosynthesis pathway of ethylene which plays an important role in adversity stress. Therefore, we used RNA sequence data to analyze their expression patterns in abiotic stresses (drought, heat, cold, and salt) and biotic stresses (powdery mildew and stripe rust) (Figure 9, Figure 10, Figure 11 and Figure 12). The results showed that *TaACS1/2/3* could respond to drought stress and cold stress and the expression of *TaACS1/3* was the highest under drought stress for 1 h and *TaACS2* for 6 h. *TaACS4/5/6* could not only respond to heat stress but also to cold stress, and the expression level of them was the highest after 6 h of heat stress. Drought, salt, and stripe rust stress could induce the high expression of TaACS7/9/10, and the expression of TaACS9 reached its highest level after 24 h of salt stress. TaACS8/11/12 showed a high expression level, which was induced by cold and stripe rust stress. These results suggest that the *TaACS* gene might play an important role in both biotic and abiotic stresses in wheat. Among these, *TaACS6* was induced under four abiotic stresses, and the amount of induction was up by several times, showing excellent tolerance.

### 2.8. Analysis of Wheat ACS Protein Interaction Network

**Figure 13:**
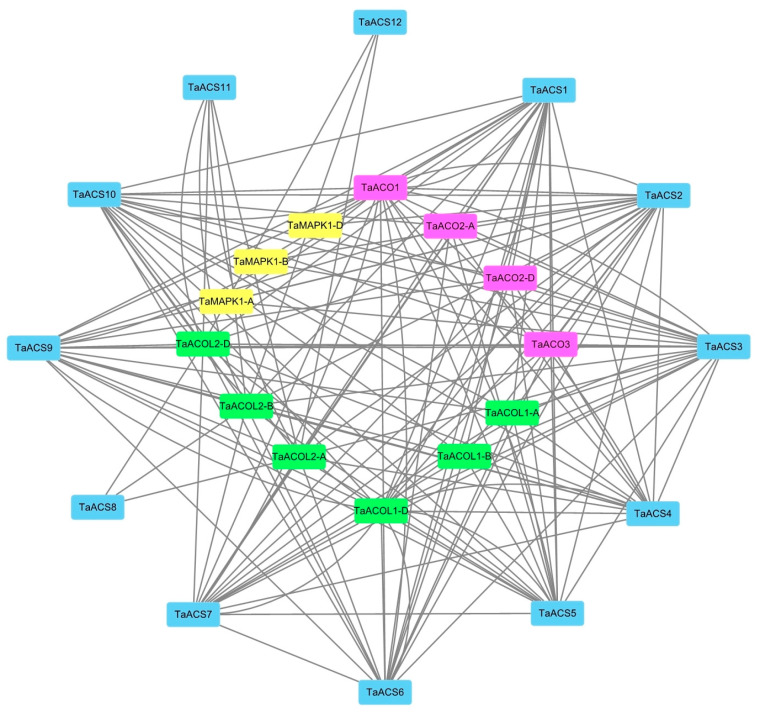
Predicted protein interaction networks of typical TaACS proteins with other wheat proteins using the STRING tool. The purple rectangle, green rectangle, and yellow rectangle represented TaACO proteins, TaACOL proteins, and MAPK proteins, respectively. The two-rectangle connected by the gray line represented the interaction between the proteins. The blue rectangle represented TaACS proteins.

Due to the incomplete database of protein interactions in wheat, a homology comparison method was used to predict the interacting proteins of TaACS. There were 13 predicted proteins interacting with TaACS, and these were named according to their homology (Figure 13 and Table S2), which was divided into three categories: ACC oxidase, AC-like oxidase, and mitogen-activated protein kinase (MAPK). In addition, we found interactions among the TaACS members. It was speculated that TaACS performed different functions by forming networks with its interacting proteins.

### 2.9. Subcellular Localization Analysis of TaACS Genes

**Figure 14:**
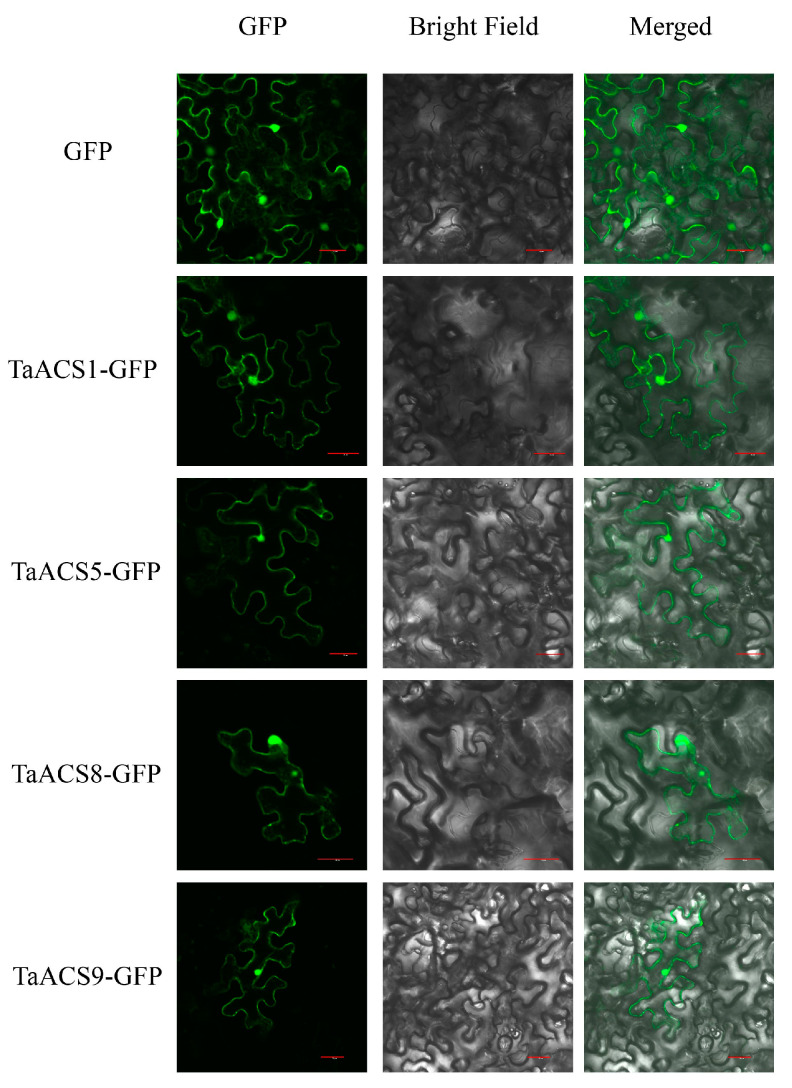
Subcellular localization of TaACS1, TaACS5, TaACS8 and TaACS9. The recombinant plasmid and control plasmid were transiently expressed in tobacco cells. The scale bar represents 30 mm.

In order to confirm whether the localization of TaACS was consistent with the predicted results, we performed subcellular localization using tobacco. The fluorescent signal was observed after 72 h of injecting Agrobacterium into tobacco leaves. As a control, the fluorescence signal was measured in the whole cell. The fusion proteins TaACS1-GFP, TaACS5-GFP, TaACS8-GFP, and TaACS9-GFP displayed green fluorescence signals on the nucleus, cytoplasm, and cell membrane, which was consistent with the predicted result (Figure 14 and Table S4). 

### 2.10. Validations of the Expressions of TaACS Genes Using RT-qPCR Analyses

**Figure 15:**
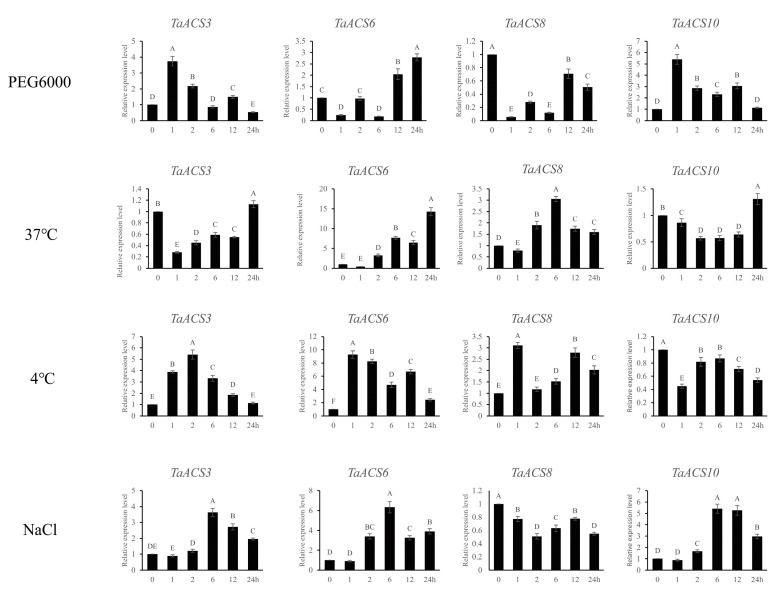
Expression analyses of typical *TaACS* genes in wheat variety Chinese Spring (CS) under different treatments by RT-qPCR. Relative expression levels of typical *TaACSs* in response to PEG6000 (20%), heat (37 °C), cold (4 °C), and NaCl (200 mM) for 0 h, 1 h, 2 h, 6 h, 12 h, and 24 h in the leaves at the two-leaf stage. Data were normalized with the *β*-*actin* gene. Vertical bars indicate standard deviations. Different capital letters indicate extremely significant differences at *p* < 0.01 according to one-way ANOVA and post hoc Tukey’s test.

Based on the expression profile, there were four candidate genes that were selected to verify the reliability of transcriptome data by RT-qPCR. It was found that the expression trend of these genes was basically consistent with RNA-seq analysis (Figure 15 and Table S4). Under simulated drought stress, the expression of *TaACS3*, *TaACS6*, and *TaACS10* were up-regulated, but the expression of *TaACS8* was down-regulated. Among them, *TaACS3* and *TaACS10* peaked at 1 h after treatment, which meant that they could belong to the early genes of drought stress. Under 37 °C of heat stress, the expression of *TaACS6* and *TaACS8* was up-regulated continuously after 2 h of treatment, which peaked at 24 h and 6 h, respectively. However, *TaACS3* and *TaACS10* were up-regulated only after 24 h of treatment. Under 4 °C of cold stress, the expression of *TaACS3*, *TaACS6*, and *TaACS8* was consistently up-regulated, while *TaACS10* was down-regulated. The expression trend under salt stress was similar to simulated drought stress, except that *TaACS8* was down-regulated, and the expression of the other three genes was up-regulated. The above results indicate that most treatments could significantly induce the *TaACS* gene, and the transcriptome data in this study were reliable.

## 3. Discussion

Ethylene plays an important role in plant growth, development, and adversity stress tolerance [1,32]. ACS is the key rate-limiting enzyme in ethylene synthesis which is encoded by a multi-gene family [15]. Translational transcription is susceptible to protein regulation as well as phosphorylation modification to determine ethylene accumulation and signal transduction, which is an important enzyme protein [23]. In this study, we identified 12 *ACS* members in wheat and comprehensively analyzed the *TaACS* gene family by their physicochemical properties, evolutionary relationships, gene structure, cis-acting elements, covariance, gene expression patterns, miRNA targeting prediction, and protein interactions. Through the RNA-Seq expression profile and qRT-PCR experiments, it was found that *TaACS6* was highly expressed under multiple stresses, showing excellent stress resistance.

Segment translocation between non-homologous chromosomes is an important form of chromosomal variation in wheat. Liu et al. (1991) determined fragment translocations between the non-homologous chromosomes 4A, 5A and 7B through the study of wheat genome [33]. King et al. (1994) found that in *Aegilops umbellulata*, *Triticum urartu*, and *Thinopyrum bessarabicum* there were also many fragment translocations on the fourth and fifth chromosomes which were proved using RFLP technology. At the same time, they were found on the fourth and seventh chromosomes in *Secale montanum* [34]. Zhang et al. (2021) had similar results in the study of the TaCAT gene family. *TaCAT1-B* and *TaCAT1-D* were located in 4B and 4D, respectively, while their homologous gene *TaCAT1-A* was located on 5A [35]. In this study, we found that the homologous genes *TaACS8*, *TaACS11* and *TaACS12* were located on chromosomes 4A, 7A and 7D, respectively. We speculated that this may be due to a non-homologous chromosomal translocation between 4A and 7B. It showed that the stability of the wheat genome was maintained by a similar variation between genomes in the process of continuous evolution and domestication, which was the basis of the formation of 21 chromosomes [36].

These roots could absorb water and nutrients from the soil and transfer them to other parts of the plant, which are important organs for growth and development [37]. Previous studies have shown that ethylene plays an important role in root development, including the formation of lateral roots and adventitious roots, the establishment of a root stem cell microenvironment, and the regulation of root hair development [10]. Takahashi et al. (2003) found that the external application of IAA and ethylene in acid increased the expression levels of *Ls-ACS1* and *Ls-ACS2*, which led to an increase in the ethylene content in seedlings to promote root hair formation [38]. A study on the spatiotemporal expression specificity of genes could provide useful information for understanding their functions, and the type of cis-acting elements in the promoter region could also reflect the function of this gene to some extent [39,40]. In this study, we analyzed the expression of *TaACS* in different tissues and different stages. All members had a high expression in the roots of seedling, the three-leaf, and flag leaf stage, except for *TaACS7/8/11/12*. This was consistent with the study on the dominant expression of *PnACS* [7]. Through promoter sequences analysis, we also found that all *TaACS* members contained W box cis-acting elements, which were related to root development. Hu et al. (2018) showed that *TaWRKY51* could regulate the expression of *ACS* genes by binding with the W box promoter in the *ACS* gene, which affected ethylene synthesis and influenced lateral root formation in wheat [24]. This indicates that *TaACSs* plays an important role in the wheat root system and could be used as a candidate gene for studying wheat root systems and selecting high-quality germplasm resources.

Usually, the gene can be regulated by interactions between multiple cis-acting elements and trans-acting elements in the region of the promoter [39]. Therefore, the analysis of gene expression under stress and in combination with the cis-acting elements of the promoters is an important reference significance for understanding the transcriptional regulation and potential function of genes. Wang et al. (2005) found that *AtACS4*, *AtACS5,* and *AtACS7* enhanced their gene expression under the hormones and stress conditions by multiple elements in their genes [41]. Zhou et al. (2022) found that many cis-acting elements were found in *TRX* genes and were induced by drought and other stresses, such as drought response elements MBS, MYB or MYC and salt stress response related elements DRE or MYB [42]. In this study, the expression of *TaACS1/3/6/7/9/10* was upregulated after drought treatment, and they contained many drought stress response elements, including MBS, MYB, or MYC. After cold stress treatment, except for *TaACS7/9/10*, other genes were upregulated, and they all contained one or more cold response elements, such as LTR or DRE. After 24 h of the salt stress treatment, the expression level of *TaACS2/3/7/9/10* was upregulated, while *TaACS8/11/6* was upregulated after 6 h of the salt stress treatment. These genes contain one or more DRE elements in the promoter regions. The above results indicated that *ACS* genes played important roles in the stress response of plants, and the different transcriptional levels of *ACS* genes could be caused by the different cis-elements of promoters. The regulatory mechanisms of the TaACS promoter region in response to different stresses need further investigation.

In addition, we also found a variety of hormone elements that regulated plant growth and development. The results showed that all members contained ABRE, CGTCA-motif, and TGACG-motif elements in response to ABA and MeJA stresses. Most members contained one or more elements in response to IAA and GA, such as P-box, TATC-box, TGA-box, and Auxin-core. A few members also contained TCA elements in response to SA. Studies have shown that Auxin can induce the expression of *ACS* genes in various plants, such as the tomato, *Arabidopsis*, and mung bean [43]. ABA regulates the activity of the ACS protein by modifying ACS phosphorylation, which affects the synthesis of ethylene to modulate root growth [38]. Exogenous JA can increase the transcriptional abundance of *ACS1* in peach fruits, regulate ethylene signaling pathways, delay the metabolism of fruit cell membranes, and reduce the chilling injury of peach fruits [44]. ABA, GA, and JA induced the expression of three *OsACS* genes [45]. These results suggested that exogenous hormones could regulate the expression of the ACS gene by binding to multiple hormone response elements in the promoter region, which can affect plant growth, development, and stress response.

MicroRNAs (miRNAs) are small non-coding RNAs that can regulate gene expression at the post-transcriptional level by inhibiting or promoting the degradation of mRNA [46]. Previous studies have shown that miRNAs regulate a variety of key biological processes, including plant growth, development, and stress responses [47], and are evolutionarily conserved in different species [48]. The miR444 played an important role in root development, tillering formation, and stress response [49] Jin et al. discovered that wheat-specific miRNA tae-miR9655 was induced by inoculation with *Fusarium graminearum* and regulated the response of *Fusarium graminearum* by inhibiting its target genes [50]. The research was conducted to predict the potential functions of genes through their target thought relationship with miRNAs. In this study, *TaACS10* was targeted by miRNAs, the tae-miR444b, tae-miR444a, and tae-miR9655-3p, including related root development, which suggested that *TaACS10* could participate in root development. Other studies have shown that tae-miR531, which has been shown to have a strong response to drought memory by regulating the expression of its target genes, and the overexpression of miRNA tae-miR531 could significantly improve the drought resistance of transgenic *Arabidopsis* [51]. In this study, tae-miR531 targeted *TaACS11*, which suggested that *TaACS11* might respond to drought stress through targeted regulation. Previous studies using microRNA sequencing and proteome analysis found that miR2275 participated in early meiosis in wheat by regulating its target gene expression [52]. In this study, tae-miR2275-3p targeted homologous genes, *TaACS8*, *TaACS11*, and *TaACS12*, which meant that they could play a role in early meiosis in wheat. Predicting miRNA-target gene relationships can provide important clues for precisely regulating the TaACS function [53].

The interactions between proteins constitute a major component of the cellular biochemical reaction network and are important in regulating cellular signal transduction. ACS and ACO are key enzymes in the ethylene biosynthesis pathway. The former is a rate-limiting enzyme, and the latter is a key enzyme for oxidizing ACC to synthesize ethylene, which jointly regulates ethylene biosynthesis by interacting with each other [19]. Wan et al. screened four ACOs, interacting with *SmACS* in eggplant [54]. Changung Park (2021) showed that ACS proteins regulated activity and stability by interacting with complex groups in subunits to form homodimers or heterodimers, which ultimately regulated ethylene biosynthesis [23]. In this study, there were two types of ACO and ACO-like interacting proteins that were predicted, which is consistent with previous research results. Therefore, we speculated that the interactions between ACS proteins could play an important role in regulating the stability and activity of ACS. Wang et al. (2022) found in *Arabidopsis* that pathogen infection activated MPK3/MPK6, which could improve the protein stability of ACS2/ACS6 through phosphorylation, thereby inducing ethylene synthesis. On the other hand, they can also activate the downstream transcription factor ERF-1A which participates in the ethylene signaling pathway and inhibits ethylene synthesis through negative feedback, thereby maintaining ethylene at an appropriate level [55]. Therefore, we speculated that the interaction between ACS and MAPK in wheat might also play a similar role. The interaction mechanisms of the above-mentioned TaACS proteins need further study.

## 4. Materials and Methods

### 4.1. Identification and Characterization of TaACS Genes

The genome-wide data of *Arabidopsis* were downloaded from TAIR10 (TAIR—Home Page arabidopsis.org). The genome-wide data of rice were downloaded from IRGSP-1.0. In the published articles related to *ACS* gene families, we obtained the IDs of 9 *AtACS* and 5 *OsACS* genes. The ACS protein sequences of *Arabidopsis* and rice were extracted by TBtools using the *AtACS* and *OsACS* gene IDs. The wheat database was searched by the blast method in Ensemble Plants, and candidate proteins of the *TaACS* gene family were screened according to their E-value ≤ 10^−10^ and a homology higher than 70%. The candidate ACS protein sequences were submitted to NCBI-CDD and SMART and analyzed as to whether they contained aminotransferase class I and II domains to confirm the integrity of the conserved domains. It was named according to its position on the chromosome. The molecular weight (MW), isoelectric point (PI), and water solubility (GRAVY) of the family members were analyzed and identified using the ExPaSy online tool. Using TBtools v1.120 to extract amino acid length information based on GFF3 files. The prediction of subcellular localization was obtained by the WoLFPSORT online tool.

### 4.2. Phylogenetic Analysis

The ACS protein sequences of *Arabidopsis*, rice, and wheat were analyzed by MEGAv5.0, and the phylogenetic tree was constructed by the Neighbor-Joining method. The Bootstrap parameter value was set to 1000, and the other default parameters were selected.

### 4.3. Gene Structure and Conserved Motif Analysis of the TaACS Gene Family

Conserved motifs of TaACS protein sequences were analyzed using MEME (https://meme-suite.org/meme/tools/meme, accessed on 11 November 2022) with a maximum number of 10 motifs to obtain data files. The structural information of exons, introns, and the UTR of *TaACS* family members was obtained from wheat GFF3. Gene Structure View (Advanced), a Tbtools software program(v3.9.1), was used for motif and gene structure visualization analysis.

### 4.4. Analysis of Chromosomal Localization and Gene Duplication Events in the TaACS Gene Family

The location Information of the *TaACSs* gene on the chromosomes was obtained from the wheat GFF3 file and visualized by Tbtools. The MCScanX program of Tbtools was used to obtain collinear files between wheat species and different species. This included wheat, *Triticum dicoccoides*, *Aegilops tauschii*, and rice. The Tbtools programs Circos and Multiple synteny plot were used to visualize the collinear. The values of the non-synonymous substitution (Ka) and synonymous substitution (Ks) of repetitive genes were calculated by the Simple Ka/Ks Calculator in Tbtools.

### 4.5. Analysis of Cis-Regulatory Elements in Promoters

The 2000 bp promoter sequence upstream of the transcriptional initiation site of *TaACS* was extracted by TBtools. We submitted to the PlantCare database (http://bioinformatics.psb.ugent.be/webtools/plantcare/html/, accessed on 21 November 2022) to predict and analyze the cis-regulatory elements of the *TaACS* promoter and then screened the cis-acting elements in relation to plant growth, development, hormone response, and stress response. Tbtools Heart Map was used for visual analysis.

### 4.6. Prediction of miRNA Target Site of TaACS

The mRNA sequences of *TaACS* were submitted to the pSNaTarget online website (https://www.zhaolab.org/psRNATarget/, accessed on 28 November 2022) for the prediction of TaACS miRNA target sites. Visual Analysis using cytoscape Software (v3.9.1).

### 4.7. Expression Pattern Analysis

From the published RNA-Seq database of Wheat URGI (https://urgi.versailles.inra.fr/files/RNASeq-Wheat/, accessed on 8 December 2022) and the NCBI Sequence Read Archive database (https://www.ncbi.nlm.nih.gov/, accessed on 8 December 2022) we downloaded available RNA sequence sample data from five tissues (root, stem, leaf, ear, and grain) and six biotic/abiotic stress conditions (drought, heat, salt, cold, powdery mildew and stripe rust) for *TaACS* expression profile analysis. Tbtools was used for the visualization of the heat map.

The material used was Chinese spring in this study, which was provided by the State Key Laboratory of Crop Stress Biology in Arid Areas and College of Agronomy, Northwest A&F University. Chinese spring wheat seedlings were placed in an incubator (23 °C, 16 h light/8 h black, relative humidity 70%), where they grew to two leaves and one heart, which were treated with 20% polyethylene glycol (PEG), 0.2 mol/L NaCl solution, 4 °C and 37 °C stress treatment. Samples were taken at 0, 1, 2, 6, 12, and 24 h after treatment and stored at −80 °C after snap-freezing in liquid nitrogen. The total RNA of the wheat samples was extracted by an extraction kit, and Cdna was synthesized by reverse transcription. The primers were designed by the software PrimerPremier6 and synthesized by Xi’an Qingke Biology Co., Xi’an, China.

THE Wheat Actin gene was used as the internal reference gene, and the Light Cycler^®^ 96 detection system (Roche) was used for QRT-PCR. PCR reaction system (20 μL): cDNA 1 μL, 2× SYBR Premix ExTaq 10 μL, 10 μmol/L forward and reverse primers 0.4 μL each, ddH2O 8.2 μL. Reaction procedure: 95 °C for 30 s; 95 °C for 10 s, 57 °C for 30 s, 40 cycles. There were 3 biological repeats in each treatment, and the relative expression was calculated by the 2^−ΔΔCt^ method.

### 4.8. Protein Interaction Network Analysis of TaACS

TaACS-interacting proteins were predicted by the STRING online tool (STRING: functional protein association networks (string-db.org). The visualization of the predicted interaction network graph was performed using Cytoscape software (v3.8.2).

### 4.9. Subcellular Localization

Four *TaACS* ORFs were inserted into the pCambia1302-GFP vector. The recombinant vector 35S: *TaACS*-GFP was transformed into the Agrobacterium tumefaciens strain GV3101. The correctly sequenced agrobacterium tumefaciens containing the recombinant vector was injected into 3–4 weeks-old tobacco leaves. The fluorescence signal was observed by a laser confocal scanning microscope (Olympus FV3000, Peking, China) 48 h later.

## 5. Conclusions

In this study, we analyzed the gene structure, conserved structural domains, evolutionary tree, and promoter cis-acting elements and predicted the subcellular localization, targeting the relationship with miRNAs and interacting proteins of the *TaACS* gene family. Finally, this was combined with RNA-Seq data, and we found that 12 *TaACS* genes had tissue-specific and responsive properties to adversity stress. The expression of four of these genes was further verified by RT-qPCR. It was found that TaACS6 showed excellent resistance to cold, heat, salt, and drought stress and could be an excellent candidate gene for resistance. We also analyzed that the homologous genes *TaACS8, TaACS11*, and *TaACS12* were located on chromosomes 4A, 7A, and 7D, respectively, probably due to non-homologous chromosomal translocations. Overall, this study lays the foundation for further understanding the role of *TaACSs* and provides valuable resistance genes for wheat breeding.

## Figures and Tables

**Table 1:** Ka/Ks of *TaACS*.

		Ka	Ks	Ka/Ks
TaACS1	TaACS2	0.007273	0.06513	0.111666
TaACS1	TaACS3	0.006363	0.071235	0.08932
TaACS2	TaACS3	0.005905	0.057409	0.102852
TaACS4	TaACS5	0.008461	0.103747	0.081557
TaACS4	TaACS6	0.004963	0.063434	0.078242
TaACS5	TaACS6	0.007459	0.064963	0.114821
TaACS7	TaACS9	0.014696	0.055432	0.26512
TaACS7	TaACS10	0.007325	0.06421	0.114077
TaACS8	TaACS11	0.018778	0.097469	0.192654
TaACS8	TaACS12	0.011384	0.096819	0.117577
TaACS9	TaACS10	0.01471	0.073606	0.199843
TaACS11	TaACS12	0.008741	0.093894	0.093091

## Data Availability

The data presented in this study are available in the article and Supplementary Materials.

## Supplementary Materials

The following supporting information can be downloaded at: https://www.mdpi.com/article/10.3390/ijms241311158/s1.
