# Supplementary material for: Genome-Wide Analysis and Identification of 1-Aminocyclopropane-1-Carboxylate Synthase (ACS) Gene Family in Wheat (Triticum aestivum L.)
Source: Int J Mol Sci. 2023 Jul 6;24(13):11158. doi: 10.3390/ijms241311158 (PMC10342151; doi:10.3390/ijms241311158)
Supplement: Supplementary file 1 [file ijms-24-11158-s001.zip › ijms-2450527-supplementary/Supplementary Figure.pdf]

```
                 *        20         *        40         *        60
TaACS01 : ------------------------------------------------------------------ :   -
TaACS02 : ------------------------------------------------------------------ :   -
TaACS03 : ------------------------------------------------------------------ :   -
TaACS04 : ------------------------------------------------------------------ :   -
TaACS05 : ------------------------------------------------------------------ :   -
TaACS06 : ------------------------------------------------------------------ :   -
TaACS07 : ------------------------------------------------------------------ :   -
TaACS08 : MSRKGDRADKKPHNPRSPRGVGGDREGGGRQQRRADGGMRIVVPLQGVVQGRGGLVLGSLIPCALF :  66
TaACS09 : ------------------------------------------------------------------ :   -
TaACS10 : ------------------------------------------------------------------ :   -
TaACS11 : MSRKGDRADKKPR-PRSPRGGGGDREGGGRQQRRADGGMRIVVPLQGVVQGRGGLVLGSLIPCALF :  65
TaACS12 : ------------------------------------------------------------------ :   -

             *        80         *       100         *       120         *
TaACS01 : ----------------------------------------------MASRSAELLSRMAAGDGHGE :  20
TaACS02 : ----------------------------------------------MASRSAELLSRMAAGDGHGE :  20
TaACS03 : ----------------------------------------------MASRSAELLSRMAAGDGHGE :  20
TaACS04 : --------------------------------------------MLLPAASSPPLSRVATSSAHGE :  22
TaACS05 : -----------------------------------------MGGMLLPAASTPPLSRVATSSAHGE :  25
TaACS06 : --------------------------------------------MLLPAASSPPLSRVATSSAHGE :  22
TaACS07 : -------------------------------------------MVNKVADEPQLLSKKAGCNTHGQ :  23
TaACS08 : YFLQLYIKRNRPPPGSPTPAAPSSASPAAAAGPLSPIHRSLSRGLLSPRALPALSARGAVVRAGDD : 132
TaACS09 : -------------------------------------------MVNKVADEPQLLSKKAGCNTHGQ :  23
TaACS10 : -------------------------------------------MVNKVADEPQLLSKKAGCNTHGQ :  23
TaACS11 : YFLQLYIKRNRPPPGSPTAASSAAPAGAAAAGPLSPIHRSLSRGLLSPRALPALSARGALVRAGDD : 131
TaACS12 : ------------------------------------------------------------------ :   -

               140         *       160         *       180         *        2
TaACS01 : NSSYFDGWKAYDMNPFHPQDNRGGVIQMGLAENQLSLDLIEEWSKAHPEAS----ICTAEGASQFK :  82
TaACS02 : NSSYFDGWKAYDMNPFHPQDNRGGVIQMGLAENQLSLDLIEEWSKAHPEAS----ICTAEGASQFK :  82
TaACS03 : NSSYFDGWKAYDMNPFHPQDNRGGVIQMGLAENQLSLDLIEEWSKAHPEAS----ICTAEGASQFK :  82
TaACS04 : DSPYFAGWRAYDDDPYDAVTNPGGVIQMGLAENQVSFDLLEGFLREHPEAAGWGGARPGSGVASFR :  88
TaACS05 : DSPYFAGWRAYDDDPYDAVTNPGGVIQMGLAENQVSFDLLEGFLREHPEAAGWGGARPGSGVASFR :  91
TaACS06 : DSPYFAGWRAYDDDPYDAVTNPGGVIQMGLAENQVSFDLLEGFLREHPEAAGWGGARPGSGVASFR :  88
TaACS07 : DSSYFLGWEEYEKNPYHPTTNPGGIIQMGLAENQLSFDLVEEWLEKNPDALG---LRRG-AASVFR :  85
TaACS08 : DSLYYAGLRRCADDPYHPASNPSGVIQLGLAENHLSLDLVREWMEEHAGPAMTPGGGDEERDLTIS : 198
TaACS09 : DSSYFLGWEEYEKNPFDAITNPSGIIQMGLAENQLSFDLVEEWLEKNPDALG---LRRG-AASVFR :  85
TaACS10 : DSSYFLGWEEYEKNPYHPTTNPGGIIQMGLAENQLSFDLVEEWLEKNPNALG---LRRG-AASVFR :  85
TaACS11 : DSLYYAGLRRCADDPYHPASNPAGVIQLGLAENHLSLDLVREWMEEHAGPAMTPGGGDEERDLTIS : 197
TaACS12 : --------------------------------MKLSLDLVREWMEEHAGPAMTPGGGAEERDLTIS :  34
                                        Box1

          00         *       220         *       240         *       260
TaACS01 : RIANFQDYHGLPEFRQAMAQFMGQVRGWKARFDPDRVVMSGGATGAQETLAFCLANPGEAFLVPTP : 148
TaACS02 : RIANFQDYHGLPEFRQAMAQFMGQVRGWKARFDPDRVVMSGGATGAQETLAFCLANPGEAFLVPTP : 148
TaACS03 : RIANFQDYHGLPEFRQAMAQFMGQVRGWKARFDPDRVVMSGGATGAQETLAFCLANPGEAFLVPTP : 148
TaACS04 : DNALFQDYHGLKTFRKAMASFMEKIRGGKVRFDPDRIVLTAGATAANELLTFILANPGDALLIPTP : 154
TaACS05 : DNALFQDYHGLKTFRKAMASFMEKIRGGKVRFDPDRIVLTAGATAANELLTFILANPGDALLIPTP : 157
TaACS06 : DNALFQDYHGLKTFRKAMASFMEKIRGGKVRFDPDRIVLTAGATAANELLTFILANPGDALLIPTP : 154
TaACS07 : ELAIFQDYHGLPAFKNALARFMSEQRDFRVAFDPSNIVLTAGATSANEALMFCLADQGDAFLIPTP : 151
TaACS08 : GLATYQPYDGILALKMALAGFMRQIMHESVSFDPSQMVITSGATPAMEILSFCIADPGNAFLVPSP : 264
TaACS09 : ELAIFQDYHGLPAFKNALARFMSEQRDYKVAFDPSNIVLTAGATSANEALMFCLADQGDAFLIPTP : 151
TaACS10 : ELAIFQDYHGLPAFKNALARFMSEQRDFRVAFDPSNIVLTAGATSANEALMFCLADQGDAFLIPTP : 151
TaACS11 : GLATYQPYDGILALKMALAGFMRQIMQESVSFDPSQMVITSGATPAMEILSFCLADPGNAFLVPSP : 263
TaACS12 : GLATYQPYDGILALKMAVAGFMRQIMQESVSFDPSQMVITSGATPAMEILSFCLADPGNAFLVPSP : 100
              Box2                                              Box3

             *       280         *       300         *       320         *
TaACS01 : YYPGFDRDCCWRSGVKLLPIECHSSNDFRITREAVVAAYEGARSSGVRVKGILITNPSNPLGTTAD : 214
TaACS02 : YYPGFDRDCCWRSGVKLLPIECHSSNDFRITREAVVAAYESARSNGVRVKGILITNPSNPLGTTAD : 214
TaACS03 : YYPGFDRDCCWRSGVKLLPIECHSSNDFRITREAVVAAYESATSNGVRVKGILITNPSNPLGTTAD : 214
TaACS04 : YYPGFDRDLRWRTGVNIVPVHCHSSNGFQVTVAALEAAYEEAAAGMSVRGVLLTNPSNPLGTTVE : 220
                                                                 Box4
```

```
TaACS05 : YYPGFDRDLRWRTGVNIVPVHCHSSNGFQVTVAALEAAYEEAAAAGMSVRGVLLTNPSNPLGTTVE : 223
TaACS06 : YYPGFDRDLRWRTGVNIVPVHCHSSNGFQVTVAALEAAYEEAAAAGMSVRGVLLTNPSNPLGTTVE : 220
TaACS07 : YYPGFDRDLKWRTGAEIVPVHCTSANGFRVTRSALDDAYRRAQKRRLRVKGVLITNPSNPLGTAVP : 217
TaACS08 : YYPGWDRDIKWRTGIELIPVPCRSTDNFNISITALEIAYNQAKKRGVRVRGVLISNPSNPTGSFVP : 330
TaACS09 : YYPGFDRDLKWRTGAEIVPVHCTSANGFRVTRSALDDAYRRAQKRRLRVKGVLITNPSNPLGTAVP : 217
TaACS10 : YYPGFDRDLKWRTGAEIVPVHCTSANGFRVTRSALDDAYRRAQKRRLRIKGVLITNPSNPLGTAVP : 217
TaACS11 : YYPGWDRDIKWRTGIELIPVPCRSTDNFNISITALEIAYNQAKKRGVRVRGVLISNPSNPTGSFVP : 329
TaACS12 : YYPGWDRDIKWRTGIELIPVPCRSTDNFNISITALEIAYNQAKKRGVRVRGVLISNPSNPTGSFVP : 166
                                                            Box4

                340         *        360         *        380         *
TaACS01 : RATLAMLATFATEHRVHLICDEIYAGSVFAKPEYVSIAEVIEHDAPGADRD----LIHIAYSLSKD : 276
TaACS02 : RATLAMLATFATEHRVHLICDEIYAGSVFAKPEYVSIAEVIEHDAPGVDRD----LIHIAYSLSKD : 276
TaACS03 : RATLAMLATFATEHRVHLICDEIYAGSVFAKPEYVSIAEVIERDAPGADRD----LIHIAYSLSKD : 276
TaACS04 : RSALEDVLDFVVRKNIHLISDEIYSGSVFAAPDLVSVAELVESRGDDVAG-----RVHIVYSLSKD : 281
TaACS05 : RSVLEDVLDFVVRKNIHLISDEIYSGSVFAAPDLVSVAELVESRGDDIAG-----RVHIVYSLSKD : 284
TaACS06 : RSVLEDVLDFVVRKNIHLISDEIYSGSVFAAPDLVSVAELVESRGDDVAG-----RVHIVYSLSKD : 281
TaACS07 : RADLEMIVDFVAAKGIHLVSDEIYSGTAFSEPGFVSVLEVLAARAPLAADYTLDDRVHVVYSLSKD : 283
TaACS08 : KQTLHDLLDFATEKNIHLISDEVFAGSTFGSGEFVSVAEVVNELEDFDRG-----RVHIIYGLSKD : 391
TaACS09 : RADLEMIVDFVAAKGIHLVSDEIYSGTAFSEPGFVSVLEVLAARAPLATEFALDDRVHVVYSLSKD : 283
TaACS10 : RADLEMIVDFVAAKGIHLVSDEIYSGTAFSEPGFVSVLEVLAARAPLAADYALDDRVHVVYSLSKD : 283
TaACS11 : KQTLRDLLDFATEKNIHLISDEVFAGSTFGSGEFVSVAEVVNELEDFDRG-----RVHIIYGLSKD : 390
TaACS12 : KQTLRDLLDFATEKNIHLISDEVFAGSTFGSGEFVSVAELVNELEDFDRG-----RVHIIYGLSKD : 227
                                                                   Box5

            400         *        420         *        440         *        460
TaACS01 : FGLPGFRVGIVYSYNDAVVACARKMSSFGLVSSQTQLFLAKMLGDEEFMSRFLRESARRLAARHEL : 342
TaACS02 : FGLPGFRVGIVYSYNDAVVACARKMSSFGLVSSQTQLFLAKMLGDEEFMARFLSESARRLAARHEL : 342
TaACS03 : FGLPGFRVGIVYSYNDAVVACARKMSSFGLVSSQTQLFLAKMLGDEEFMARFLRESARRLAARHEL : 342
TaACS04 : LGLPGFRVGVVYSYNDAVVTAARRMSSFTLVSSQTQKTLAAMLSDAAFAQEYIRTNRERLRVRHDH : 347
TaACS05 : LGLPGFRVGVVYSYNDAVVTAARRMSSFTLVSSQTQKTLAAMLLDAAFAQEYIRTNRERLRVRHDH : 350
TaACS06 : LGLPGFRVGVVYSYNDAVVTAARRMSSFTLVSSQTQKTLAAMLSDAAFAQEYIRTNRERLRVRHDH : 347
TaACS07 : LGLPGFRVGAIYSSNPAVVSAATKMSSFGLVSSQTQYLLAALLGDKDFTRRYLAENKRRIKERHDQ : 349
TaACS08 : LSLAGFRVGVIYSYNESIVEAAAKIARFSSVSTPTQRLLVAMLSDQKFISNYLKVNRERLRKAYNL : 457
TaACS09 : LGLPGFRVGAIYSSNPAVVSAATKMSSFGLVSSQTQYLLAALLGDKDFTRRYLAENKRRIKERHDQ : 349
TaACS10 : LGLPGFRVGAIYSSNAAVVSAATKMSSFGLVSSQTQYLLAALLGDKDFTRRYLAENKRRIKERHDQ : 349
TaACS11 : LSLAGFRVGVIYSYNESIVEAAAKIARFSSVSTPTQRLLVAMLSDQKFISNYLKVNRERLRKAYNL : 456
TaACS12 : LSLAGFRVGIIYSYNESIVEAAAKIARFSSVSTPTQRLLVAMLSDQKFISNYLKVNRERLRKAYNL : 293
             Box5                     Box6

                 *        480         *        500         *        520
TaACS01 : FTSGLREVGIGCLGGNAGLFSWMDLRGMLR---EKTAEAELELWRVIIRKVKLNVSPGTSFHCGEP : 405
TaACS02 : FTSGLREVGIGCLGGNAGLFSWMDLRGMLR---EKTAEAELELWRVIIRKVKLNVSPGTSFHCREP : 405
TaACS03 : FTSGLREVGIGCLGGNAGLFSWMDLRGMLR---EKTAEAELELWRVIIRKVKLNVSPGTSFHCGEP : 405
TaACS04 : MVAGLARSGVPCLPSNAGLFVWMDMRRLLRDGGEATVADELRLWDLMLREVKLNISPGSSCHCSEP : 413
TaACS05 : MVAGLARSGVPCLRSNAGLFVWMDMRRLLGDGGEATVADELRLWDLMLHEVKLNISPGSSCHCSEP : 416
TaACS06 : MVAGLARSGVPCLPSNAGLFVWMDMRRLLGDGGEATVANELRLWDLMLHEVKLNISPGSSCHCSEP : 413
TaACS07 : LVDGLKEIGIACLESNAGLFCWVNMSHLMH---ARSFEGEMTLWKKVVFDVGLNISPGSSCHCSEP : 412
TaACS08 : LVDALKQVGIECFKSSGGFYCWADMSKFIR---SYSEKGERRLWDRLLEEAKVNVTPGSSCHCIEP : 520
TaACS09 : LVDGLKEIGIACLESNAGLFCWVNMSHLMH---TRSFEGEMTLWKKVVFDVGLNISPGSSCHCSEP : 412
TaACS10 : LVDGLKEIGIACLESNAGLFCWVNMSHLMH---ARSFEGEMTLWKKVVFDVGLNISPGSSCHCSEP : 412
TaACS11 : LVDALKQVGIECFKSSGGFYCWADMSKFIR---SYSEKGERSLWDRLLEEAKVNVTPGSSCHCIEP : 519
TaACS12 : LVDALKQVGIECFKSSGGFYCWADMSKFIR---SYSEKGERRLWDRLLEEAKVNVTPGSSCHCIEP : 356
                                                                    Box7

            *        540         *        560         *        580         *
TaACS01 : GWFRVCHANMDDETMGVALSRIRDFVRQHQQQKAKAQRWAARSHLHLSLQRHGAMASQYHALSSPM : 471
TaACS02 : GWFRVCHANMDDETMGVALNRIRDFVRQHQQQKAKAQRWAARGHLHLSLQRHGAMASQYHALSSPM : 471
TaACS03 : GWFRVCHANMDDETMGVALSRIRDFVRQHQQQKAKAQRWAARGHLHLSLQRHGAMASQYHALSSPM : 471
TaACS04 : GWFRVCFANMSLDTLDVALARMSRFMDRWN----KAT----------TLQEQH*------------ : 452
TaACS05 : GWFRVCFANMSLDTLDVALARMSRLMDRWN----KAT----------TLQEQH*------------ : 455
TaACS06 : GWFRVCFANMSLDTLDVALARMSRFMDRWN----KAT----------TLQEHH*------------ : 452
TaACS07 : GWFRVCFANMSAKTLDVAMQRLREFVQTCSFKAAPAALR--------RAAGPARSMSCPLAMNMKW : 470
TaACS08 : GWFRCCFTTLRERDIPVVVERLRRVTASHKSNR*-------------------------------- : 553
TaACS09 : GWFRVCFANMSAKTLDVAMQRLGEFVQTSSCKAAPAALR--------RAAGPARSMSCPLAMNMKW : 470
TaACS10 : GWFRVCFANMSAKTLDVAMQRLGGFVQTSSCKAAPAALR--------RAAGPARSMSCPLAMNMKW : 470
             Box7
```

```
TaACS11 : GWFRCCFTTIREQDIPVVVERLRRITDGHKSNC*-------------------------------- : 552
TaACS12 : GWFRCCFTTIREQDIPVVVERLRRVTDGHKSNR*-------------------------------- : 389
             Box7

             600        *
TaACS01 : AALLSPQSPLVHAAS* : 486
TaACS02 : AALLSPQSPLVHAAS* : 486
TaACS03 : AALLSPQSPLVHAAS* : 486
TaACS04 : ---------------- :   -
TaACS05 : ---------------- :   -
TaACS06 : ---------------- :   -
TaACS07 : ALRLTPGFADRKAER* : 485
TaACS08 : ---------------- :   -
TaACS09 : ALRLTPSFADRKAER* : 485
TaACS10 : ALRLTPGFADRKAER* : 485
TaACS11 : ---------------- :   -
TaACS12 : ---------------- :   -
```

Figure S1. Multiple alignment of the domains from the TaACS proteins based on the genedoc software. The seven highly conserved domains of the ACS isozymes are marked as boxes 1–7. * represents the omitted amino acid sequence number. Green represents hydrophobic amino acids, red represents charged amino acids, and blue represents polar amino acids.
